# Proceedings of the 2nd Implementation Science Health Conference Australia: Sydney, NSW, Australia, 23-24 March 2023

**DOI:** 10.1186/s13012-023-01292-1

**Published:** 2023-09-07

## Mitchell Sarkies^1 2 a^, Hossai Gul^3 4 a^, Lisa Pagano^5^, Tess Aungles^2^, Meghan Finch^6^, on behalf of the ISHCA Scientific Committee^b^

### ^1^ Sydney School of Health Sciences, Faculty of Medicine and Health, The University of Sydney; ^2^ Implementation Science Academy, Sydney Health Partners; ^3^ Implementation Lab, TD School, University of Technology Sydney; ^4^ Implementation Science Platform, Maridulu Budyari Gumal (SPHERE); ^5^ Centre for Healthcare Resilience and Implementation Science, Australian Institute of Health Innovation, Faculty of Medicine, Health and Human Sciences, Macquarie University; ^6^ National Centre of Implementation Science, School of Medicine and Public Health, University of Newcastle

*Implementation Science 2023,* **18(2):**

^a^ Mitchell Sarkies and Hossai Gul contributed equally to this paper

^b^ Andrew Baillie, Lauren Christie, Caleb Ferguson, Julie Gawthorne, Helen Goldsmith, Nicolette Hodyl, Natasha Howard, Karen Hutchinson, Marlena Klaic, Joanna Moullin, Nicole Nathan, Sanne Peters, Nicole Rankin, Ben Smith, Serene Yoong.

**I1 Introduction**

In March 2023, the theme of the 2nd Implementation Science Health Conference Australia (ISHCA) was ‘Scale. Sustain. Success.’ ISHCA has evolved from an annual Implementation Science Symposium, initially hosted by Sydney Health Partners, into a national conference co-hosted by several notable entities concerned with the translation of evidence-based innovation into practice; including the National Health and Medical Research Council (NHMRC) accredited Research Translation Centres, NHMRC funded Centres of Research Excellence, health and medical research institutes, and government health agencies. The conference aimed to enhance the translation, implementation and impact of health and medical research by advancing the field of implementation science and practice within the Australian health care system. Leaders in implementation science and practice were brought together to share successful innovations and learnings on how to implement and sustain evidence-based healthcare improvement at scale. Representatives from clinical practice, academia, management, and policy helped shape the conversations to define the future of implementation science, by hearing from expert speakers, joining conversations, and connecting with colleagues and peers. This supplement summarises the conference proceedings and includes the peer-reviewed abstracts presented.

ISHCA is a key national collaborative partnership that transcends Australian state and territories to improve systems of healthcare delivery. ISHCA was co-hosted by NHMRC accredited Research Translation Centres: Sydney Health Partners, Maridulu Budyari Gumal (SPHERE), NSW Regional Health Partners, Melbourne Academic Centre for Health, Monash Partners, and Health Translation SA; NHMRC funded Centres of Research Excellence: National Centre of Implementation Science; health and medical research institutes: South Australian Health and Medical Research Institute; and government health agencies: NSW Agency for Clinical Innovation. NHMRC accredited Research Translation Centres are recognised leading centres of collaboration in Australia that provide excellence in research, translation, partnerships, and evidence-based training of health professionals and other healthcare end-users such as consumers.

Almost 300 delegates joined the face-to-face conference in Sydney, Australia on the 23-24 March 2023 from a diversity of healthcare settings, including representation from clinicians (22%) and students (10%). National representation was achieved, with attendees from across states and territories: New South Wales (77%), Victoria (13%), Queensland (3%), South Australia (3%), Australian Capital Territory (2%), Western Australia (1%), Tasmania (0.4%), and international (1%).

The first day of the conference opened with a plenary session entitled ‘Implementation sustainability and scaling: achievements and future directions in Australia and internationally’. Associate Professor Rachel Shelton discussed the conceptualisation of implementation sustainability as a dynamic and multidimensional process, followed by Professor Luke Wolfenden who shared insights into the impact of scaling up interventions, allowing for adaptations to interventions, and future approaches to scale up. Building on the keynote presentations, two subsequent invited speaker sessions further delved into the topics of ‘Implementation sustainability’ and ‘Implementation scale up’. The first session on implementation sustainability featured leading experts Professor Jill Francis, Dr Nicole Nathan, and Dr Joanna Moullin. The discussion included academic perspectives on a research agenda for three levers for implementation sustainability: implementation strategies, implementability of interventions, and contextual circumstances. This was followed by public health insights into the importance of implementation sustainability for delivering impact in health and medical research, and finally, a clinician-researcher discussion was held on the topic of embedding researchers into health services to implement improvements sustainably. The second session on implementation scale up featured Associate Professor Serene Yoong, Associate Professor Denise O'Connor, Professor Adrian Bauman, Dr Jackie Yeoh and Dr Ecushla Linedale. The discussion brought together experiences from scaling up implementation using trials via multiple modalities, natural experiments that are aligned with the dynamic complex nature of health systems, and partnerships between health services providers, implementation researchers, and policy makers as foundations for scale.

The second day of the conference began with a plenary session titled ‘Implementation success: opportunities for rapid implementation and new ways of working for clinician researchers’. Professor Sandy Middleton shared their experience delivering implementation research in stroke care over the past 20 years, sustaining and scaling up interventions nationally and internationally. Canadian implementation science expert, Professor Jeremy Grimshaw, followed with a vision for building the implementation science community in Australia, and the need for implementation labs as catalysts for testing implementation strategies more effectively. Annette Ruhotas rounded off the plenary session with a consumer representative perspective on how consumer engagement can be used as an implementation strategy to advocate for implementing new research findings into routine clinical care. The second day concluded with an invited panel discussion on ‘Implementation success and sustaining change at scale’ with Dr Jean-Frédéric Levesque, Adjunct Associate Professor Jo Mitchell, Professor Brendan McCormack, and Associate Professor Michelle Barakat-Johnson. The discussion included a variety of audience-led topics including: the relationship between workforce (including turnover rates) and implementation, sustaining and scaling improvements already existing within the health system, bridging the gap between academic and clinician-researchers to enhance collaborations, implementation science capability building, the need for strong consumer engagement, and diverse career pathways into implementation science and practice.

Day two began with a breakfast workshop focused on an introduction to the Behaviour Change Wheel, conducted by clinician-researchers Dr Lauren Christie, Professor Natasha Lannin, and Dr Tamina Levy. This workshop provided those new to implementation science and practice, an introduction to a practical tool, and an opportunity to meet fellow experienced researchers. Positive feedback after the workshop suggested there is a preference among participants for longer workshops in future conferences to allow time for hands-on application of new tools. Day 2 also consisted of a lightly facilitated clinician-researcher networking session. This provided dedicated, supported time and space for clinicians and researchers to discuss potential collaborations and partnerships via a round-robin method.

The Scientific Committee welcomed 112 abstract submissions, including for oral, rapid fire, and poster presentations spread across six topics:

- Building implementation capacity for program scale-up
- Ensuring public health interventions meet different community needs
- Successfully implementing novel practices in health care
- Reducing practice variation in health care
- Sustaining change in health service processes and pathways
- What works, why and for whom when implementing programs in primary care and community settings

The Scientific Committee acknowledged the diverse terms and definitions of ‘implementation science’, ‘knowledge translation’, and more broadly, ‘research translation’. Submitted abstracts were reviewed independently by two members of the Scientific Committee based on 1) whether the abstract was suitable for a presentation at ISHCA, 2) alignment with conference themes, 3) overall scientific quality, and 4) recommended presentation format. Reviewers also had the opportunity to provide additional comments. The cut-off score for inclusion was based on the number of places available for talks, and a separate cut-off score was also used for poster presentation acceptance. Accepted presentations were then reviewed by the Chair of the Scientific Committee to ensure representation from different clinical and academic backgrounds, organisations, and states and territories, with only modest changes made to the program. This supplement is arranged according to the six above-mentioned topics and includes 44 abstracts from the concurrent oral and rapid-fire presentations. The 43 poster presentations are not included in this supplement, but can be viewed here: https://ishca.au/.

Three categories of clinician, early career researcher, and student meritorious awards were awarded based on the highest rated abstract reviews. Meritorious awards were also awarded for oral and rapid-fire presentations in each concurrent session, judged by members of the Scientific Committee using a scoring metric. People’s choice awards were also awarded for oral and rapid-fire presentations in each concurrent session, based on voting from the audience. Finally, the top three voted posters (according to attendees) were invited to perform a rapid-fire presentation of their poster to the delegates prior to the concluding remarks of the conference.

We are pleased to have welcomed attendees to our face-to-face event in Sydney, after a previous virtual conference (due to COVID restrictions). The dissemination of conference abstracts facilitates the promulgation of implementation science knowledge within the Australian healthcare sector, by expanding the accessibility of conference materials in an open access publication, and the conference website to those who were unable to attend. We look forward to the 3rd Implementation Science Health Conference Australia.

## Building implementation capacity for program scale up

### O1 Sustaining health promotion interventions in real-world population settings

#### Melanie Crane

##### The University of Sydney, NSW, Australia

*Implementation Science 2023,* **18(2):**O1

**Background**

Our understanding of what determines the sustainability of health interventions is derived primarily from studies of interventions in single settings and evaluated within 1-2 years of dissemination. This poorly depicts population health interventions and a long-term goal of sustainment. To improve implementation and sustainability we examined aspects of real-world population health interventions.

**Methods**

We used a systematic approach to identify interventions that have been disseminated to prevent chronic diseases through physical activity, nutrition or obesity prevention. We reviewed 90 population health interventions, using publicly available information to gather data on theoretical determinants of sustainment (intervention characteristics, provider level and delivery level factors, and the macro external environment).

**Results**

The interventions were from English-speaking countries, delivered in various settings (schools, workplaces, community, etc) and had been sustained for 4-37 years (average 15 years). Those sustained for >15 years were more likely to be behaviour skills programs (p=0.006); and characterised as interactive innovations (i.e. face-to-face or digital) (p=0.07), had undergone modification (p=0.069). While interventions were mainly government funded (n=45; 54.9%), longer sustained programs were more likely commercially funded (p=0.053); and less likely delivered through partnerships (p=0.071) or community involvement (p=0.011).

**Conclusions**

Factors contributing to an intervention’s sustainment may change over time. Current understanding of what sustains interventions may only be relevant for the initial maintenance and lesser influence on its longer-term sustainment. Differences exist across countries, suggesting the importance of external, cultural and policy environments support of population health interventions.

### O2 Understanding the role of blended facilitation to drive quality improvement in delirium practices

#### Lucylynn Lizarondo, Alexa McArthur

##### Joanna Briggs Institute, University of Adelaide, SA, Australia

*Implementation Science 2023,* **18(2):**O2

**Background**

Facilitation has been described as both an individual role and a multifaceted process that enables individuals or teams to incorporate evidence into clinical practice and make changes to healthcare processes and outcomes. The aim of the study was to examine the internal and external facilitation activities that occurred during an evidence implementation initiative aimed at improving delirium practices in a tertiary hospital in New South Wales. We identified the specific facilitation activities performed from pre-implementation to implementation, and evaluation of processes and outcomes.

**Methods**

Using the JBI approach to evidence implementation, 11 wards/units in the hospital conducted an evidence implementation project to improve delirium screening, assessment, prevention, and management. We followed the sites as they planned, developed, and implemented their action plan to improve delirium practices in the hospital. We documented the meetings of the facilitators and held round table discussions following the pre-post period of the implementation project.

**Results**

Facilitation occurred internally via the delirium clinical champions and externally through implementation researchers who reviewed the evidence on delirium. Internal facilitation activities included education to staff, assessment of local practice through audits and barrier analysis, evaluation of practice change and peer support. External facilitation included provision of technical support and education on implementation science.

**Conclusions**

Internal and external facilitation activities were integral to the quality improvement of delirium practices. Blended facilitation may be an effective strategy to advance organisational capacity for evidence implementation and continuous quality improvement.

### O3 Scaling and sustaining a successful delirium prevention program (Eat Walk Engage): strategies and challenges experienced in a 3-year state-wide implementation program

#### Prue McRae^1^, Angela Byrnes^1^, Karen Lee-Steere^1^, Margaret Cahill^1^, Adrienne Young^1,2^, Alison Mudge^1,3^

##### ^1^Royal Brisbane and Women's Hospital, QLD, Australia; ^2^Centre for Research in Geriatric Medicine, University of Queensland, QLD, Australia; University of Queensland Medical School, QLD, Australia

*Implementation Science 2023,* **18(2):**O3

**Background**

Sustainment of effective health program is critical for long-term return on investment. Eat Walk Engage is an evidence-based delirium prevention program scaled up across Queensland public hospitals since 2019. The program facilitates continuous improvement in care of older people and sustainment is viewed as a continuing process.

**Methods**

Reflecting on program implementation and maintenance (sustainment) over 2019-2022 using the RE-AIM framework, we will describe the implementation and adoption of this ward-based program and how evidence-based factors for sustainment have been operationalised.

**Results**

Scale-up to 40 wards has been supported by a state-wide program management team with content and implementation expertise, in a phased approach. Costs of site implementation and state-wide team are supported by the Queensland Department of Health within a larger priority work program. Expert facilitators within the state-wide team train and mentor embedded facilitators and multi-professional assistants at each site. Key process measures are collected annually, with data management, site reporting and benchmarking supported by the state-wide team. Site visits, a virtual community of practice and regular teleconferences nurture relationships with opinion leaders and champions of multiple disciplines and levels. Reporting to a state-wide clinical network and an engaged Consumer Response Team supports organisational and community visibility. Challenges include high facilitator, champion and clinical lead turnover and adapting to dynamic contexts (such as the COVID-19 pandemic).

**Conclusions**

Sustaining a complex program at scale requires dedicated time and skills to maintain program visibility, develop rich relational networks across traditional governance structures, support staff and adapt to dynamic contexts.

### O4 Building capacity to foster research translation: the Sydney Health Partners Implementation Science Program 2017-2021

#### Nicole Rankin^1^, Tess Aungles^2,3^, Don Nutbeam^2,3^, Bandana Saini^4^, Merran Findlay^5^, Adrian Bauman^6^, Andrew Baillie^3,5^

##### ^1^The University of Melbourne, VIC, Australia; ^2^Sydney Health Partners, Sydney, NSW, Australia; ^3^The University of Sydney, Sydney, NSW, Australia; ^4^School of Pharmacy, The University of Sydney, NSW, Australia; ^5^Sydney Local Health District, NSW, Australia; ^6^Sydney School of Public Health, The University of Sydney, NSW, Australia

*Implementation Science 2023,* **18(2):**O4

**Background**

Capacity building in Implementation Science requires strategic programs to foster growth of the discipline. A primary goal of capacity building is to prepare the research and clinical workforce in “how to” effectively implement robust interventions and scale up initiatives in practice. This presentation will describe Sydney Health Partners multi-faceted and strategic approach to building capacity.

**Methods**

Sydney Health Partners is an Australian Health Research Translation Centre, which invested in implementation science capacity building since its inception. We used the RE-AIM framework to report on program strategy, evaluation activities and reflect on lessons learned. We report on quantitative and qualitative evaluation data collected over a five-year period.

**Results**

Reach: our team invited international experts to lead annual Symposia and Conferences (>1000 participants) and established a state-wide Community of Practice (>640 members). Effectiveness: we delivered annual Masterclasses (>100 participants) and formed strategic alliances with government agencies and other Research Translation Centres. Adoption: we funded a Research Translation Fellowship Program to embed clinician-researchers across health services. Implementation: we established a pilot and seed grants program (>15 projects funded) to foster collaborative team research. Maintenance: we established an Academic Implementation Science Network to build capacity across the University of Sydney and Local Health Districts.

**Conclusions**

This strategic, multi-faceted program has built on international capacity building programs to address a significant demand for local offerings in Implementation Science. The program foundations are in place to engage clinician-researchers in implementation research and to measure its impact in the future.

### O5 Strategies for sustainability: Implementation insights for long-term sustainment of programs that routinely collect Patient Reported Outcomes in cancer care clinical settings

#### Carolyn Mazariego^1^, Monica Krzyzanowska^2^, Mike Lovas^3^, Geoff Delaney ^4^, Michael Jefford^5^, Raymond Chan^6^, Antoinette Anazodo^7^, Bena Brown^8^, Lesley Millar ^9^, Natasha Roberts^10,11^, Bogda Koczwara^12,13^

##### ^1^School of Population Health, Faculty of Medicine and Health, University of New South Wales, NSW, Australia; ^2^ Clinical Research Unit, Princess Margaret Cancer Centre, Toronto, ON, Canada; ^3^Cancer Digital Intelligence Unit, Princess Margaret Cancer Centre, Toronto, ON, Canada; ^4^Ingham Institute for Applied Medical Research, UNSW, Liverpool, NSW, Australia; ^5^Department of Health Services Research, Peter MacCallum Cancer Centre, Melbourne, VIC, Australia; ^6^Caring Futures Institute, College of Nursing and Health Sciences, Flinders University, Adelaide, SA, Australia; ^7^Kids Cancer Centre, Sydney Children’s Hospital, Randwick, NSW, Australia; ^8^Southern Queensland Centre of Excellence in Aboriginal and Torres Strait Islander Primary Healthcare, Metro South Health, QLD, Australia; ^9^ Medical School, University of Western Australia, Perth, WA, Australia; ^10^University of Queensland Centre for Clinical Research, Herston, QLD, Australia; ^11^Metro North Health Service, QLD, Australia; ^12^Department of Clinical Oncology, Flinders Medical Centre, Flinders Health; ^3^Medical Research Institute, Flinders University, Adelaide, SA, Australia

*Implementation Science 2023,* **18(2):**O5

**Background**

In order to promote longevity of health innovations, sustainability factors need to be understood. Despite the known evidence-based benefits of routinely collecting Patient Reported Outcomes (PROs) in cancer care clinical services, implementation has been uncoordinated and inconsistent in Australia. In Canada, PRO collections have been routine practice for 10+ years. As such, this study aimed to detail influencing factors that contribute to the sustainability of this health intervention (routine collection of PROs) in health services.

**Methods**

Key stakeholders in PRO collection programs across the 14 cancer centres in the province of Ontario, Canada were invited to participate in a qualitative interview. Using an implementation science-based approach through process mapping and semi-structured sustainability-focused interviews we explored factors influencing program longevity. The interview guide was designed using the domains of the Program Sustainability Assessment Tool. Influencing barriers and enablers were identified across recommendations for sustainability and development of an evolving sustainability framework.

**Results**

Twenty-four key stakeholders were interviewed. Recommendations to implement and sustain PRO collection programs were identified across four major themes which set out the evolving sustainability framework; planning for success, acute necessities, ironing out issues, long-term essentials. Key recommendations included provision of a PRO implementation coordinator, selection of action-based outcome and process measures, and reiteration and feedback.

**Conclusions**

This study provides sustainability recommendations and insights from a mature PRO collection program. These insights, along with patient perspectives, should be considered in Australian efforts to implement similar PRO collection programs within cancer care clinical services.

### O6 Relationships, facilitation and framing: Real-world scale up

#### Angela Melder, Mandy O'Connor

##### Health and Social Care Unit, Monash University, VIC, Australia

*Implementation Science 2023,* **18(2):**O6

**Background**

The divide in implementation science between implementation research and implementation practice is a challenge. Constructs and processes where this divide is most prominent is in understanding trust and scale-up. Trust has been described as a critical factor for achieving implementation results and further research is required to explore trust in association with implementation strategies and outcomes, and, how practitioners foster and deepen trust among implementation stakeholders. A lack of scientific knowledge about scaling has clearly been articulated. While several scaling models exist, there is a persistent inadequacy in scaling improvements across health and social care system. We aimed to examine the prevailing constructs that emerge when undertaking scale-up in the real-world setting.

**Methods**

We applied an integrated knowledge translation model, whereby we partnered with implementation stakeholders to implement a complex, state-wide, Early Parenting Outcomes Framework intervention into practice. Methods included theory-driven implementation approaches with action-research methods.

**Results**

Our results provided insights into key constructs that lead to contexts that are conducive to productive scale-up processes. This includes: the need for developing relationships through prior consultation and development of the Outcomes Framework; facilitation performed by skilled Implementation Practitioners who ensure the development of trusting relationships, and leveraging a context framed within a staged approach to implementing across sites working with a broad, long-term vision of system change.

**Conclusions**

Foster relationships, sponsor skilled facilitation and frame system scale-up.

### O7 Health facility readiness for implementation of an intravenous iron intervention for pregnant women with anaemia in the Bangladesh government health system

#### Ebony Verbunt^1^, Bidhan Sarker^2^, Quaiyum Rahman^2^, Shamim Ahmed ^2^, Imrul Hasan^2^, Jena Hamadani^2^

##### ^1^Melbourne School of Population and Global Health, University of Melbourne, VIC, Australia; ^2^International Centre for Diarrhoeal Disease Research, Maternal and Child Health Division, Bangladesh

*Implementation Science 2023,* **18(2):**O7

**Background**

Bangladesh provides oral iron and folic acid supplementation for treatment of antenatal anaemia. However, due to low coverage and adherence to oral iron, it continues to remain a significant public health problem. An alternative treatment for antenatal anaemia is modern intravenous (IV) iron. IV iron is not available in the government health system. By conducting a health facility readiness assessment, we aim to ascertain the availability of resources required to screen for antenatal anaemia and administer IV iron, and to inform understanding of scalability of the intervention.

**Methods**

An interview-administered questionnaire adapted from the WHO Service Availability and Readiness Assessment Standard tool was used to collect data from the 22 health facilities in Bandar Upazila. Data were verified by structured facility observation. Service readiness for anaemia screening was determined by mean readiness index (RI) scores across seven domains, as was service readiness for IV iron administration. Data analysis was conducted using Stata 16.

**Results**

Community Clinics had the lowest RI score for anaemia screening (15%), followed by UH&FWC and Union Health Sub Centre (UHSC) (50%), and Upazila Health Complex (UHC), Mother and Child Welfare Center (MCWC), and District Hospital (DH) (95%). For IV iron administration UHC, MCWC, and DH had the highest RI score (90%) compared to UH&FWC/UHC (40%).

**Conclusions**

Health facilities are not “ready” to screen for anaemia and administer IV iron. Healthcare providers will require guidelines and training. Haemoglobin screening tools are required, particularly for CCs, and equipment and medicines for IV iron administration at UH&FWCs/UHSCs.

## Ensuring public health interventions meet different community needs

### O8 “Re-implementing” Parent-Child Interaction Therapy (PCIT) in Aotearoa / New Zealand

#### Melanie Woodfield^1,2^, Sally Merry^1^, Sarah Hetrick^1^

##### ^1^University of Auckland, Auckland, New Zealand; ^2^Te Whatu Ora (Health New Zealand), New Zealand

*Implementation Science 2023,* **18(2):**O8

**Background**

Parent-Child Interaction Therapy (PCIT) is an effective treatment for childhood conduct problems. Sustainment of evidence-based interventions in routine care environments is notoriously challenging, and relatively few clinicians deliver PCIT, despite having received intensive training. The aim of this research programme was to systematically develop and pilot a multi-component “re-implementation” intervention, targeting PCIT-trained clinicians who are not, or are rarely, using PCIT.

**Methods**

A series of sequential mixed methods studies included: 1) a systematic review of existing PCIT implementation interventions; 2) a cross-sectional survey of PCIT-trained clinicians to explore implementation determinants; 3) focus groups with clinicians, managers and funders to prioritise determinants and shape an intervention and; 4) a randomised, controlled feasibility trial of this intervention.

**Results**

1) The systematic review suggested that little research has explored the sustainment of PCIT. 2) Survey outcomes suggested that PCIT-trained clinicians view PCIT as both acceptable and effective, but barriers included lacking suitable equipment and PCIT-trained colleagues, and concerns about the use of time-out with children. 3) Analysis of focus group data utilising the Theoretical Domains Framework allowed for specification of hypothesised mechanisms of action of the proposed intervention components, which will be described, along with 4) preliminary results from the PCIT re-implementation pilot trial.

**Conclusions**

Where implementation has stalled or languished, “re-implementation” may be possible, and makes good sense fiscally and practically. Successful re-implementation requires a systematic exploration of context-specific determinants of practice, followed by theory-driven understanding of mechanisms of action to inform, prioritise and refine selection of intervention components.

### O9 Assessing the scalability of evidence-based healthy eating and physical activity interventions in early childhood education and care services across Australia

#### Alice Grady^1,2,3,4^, Jacklyn Jackson^1^, Melanie Lum^1,2,3^, Sze Lin Yoong^2,5^

##### ^1^School of Medicine and Public Health, University of Newcastle, NSW, Australia; ^2^Population Health, Hunter New England Local Health District, NSW, Australia; ^3^Public Health Program, Hunter Medical Research Institute, NSW, Australia; ^4^National Centre of Implementation Science, University of Newcastle, NSW, Australia; ^5^Global Centre for Preventive Health and Nutrition, Institute for Health Transformation, Deakin University, VIC, Australia

*Implementation Science 2023,* **18(2):**O9

**Background**

Despite public health and research investment, few early childhood education and care (ECEC)-based obesity prevention programs are implemented at scale. To maximise public health impact, interventions need not only be effective, but consider a range of scalability factors, such as reach, cost, end-user infrastructure, and local context. This study describes perceptions regarding the scalability of evidence-based healthy eating and physical activity interventions among Australian ECECs; and associations between intervention scalability and service characteristics.

**Methods**

453 ECECs across Australia completed a cross-sectional survey assessing the scalability of six evidence-based healthy eating (e.g. training educators to support child healthy eating), and six evidence-based physical activity (e.g. providing sufficient opportunities for child physical activity) interventions using items based on the Intervention Scalability Assessment Tool. Linear regression analyses were used to explore associations between intervention scalability and service characteristics.

**Results**

The mean scalability score for all interventions was 40.18 (out of 50). The highest-scoring healthy eating and physical activity interventions were ‘providing healthy eating education and activities for children’, and ‘providing sufficient opportunities for child physical activity’, respectively. The lowest scoring were ‘providing families with lunchbox guidelines’ and ‘engaging families in activities to increase child physical activity’. Services located in higher SES areas scored the scalability of ‘having a physical activity policy’ significantly higher than lower SES services; larger services, scored the scalability of ‘training educators to support child healthy eating’ significantly higher than smaller services; and services located in rural areas, scored the scalability of ‘making healthy menu modifications’ significantly lower than urban services.

**Conclusions**

Findings indicate number of healthy eating and physical activity interventions in ECEC are both evidence-based and scalable, and as such warrant wide spread implementation. Investigation into lower scoring interventions among smaller ECECs, and those located in rural and lower SES areas is warranted.

### O10 Cracks in the Ice: A digital toolkit that improves attitudes and knowledge about crystal methamphetamine

#### Anna Grager^1^, Steph Kershaw^1^, Louise Birrell^1^, Katrina Champion^1^, Hannah Deen^1^, Felicity Duong^1^, Lexine Stapinski^1^, Nicola Newton^1^, Frances Kay-Lambkin^2^, Maree Teesson^1^, Cath Chapman^1^

##### ^1^The Matilda Centre for Research in Mental Health and Substance, University of Sydney, NSW, Australia; ^2^Hunter New England Research Institute, NSW, Australia

*Implementation Science 2023,* **18(2):**O10

**Background**

Cracks in the Ice (cracksintheice.org.au, CITI) is a digital initiative aiming to develop and disseminate evidence-based resources about crystal methamphetamine for people who use crystal methamphetamine, their families, health workers and communities. An online survey was conducted from February 2022 to April 2022 to evaluate whether CITI was meeting the needs of the community, as well as whether the redesign of the toolkit in 2021 improved site usability and engagement.

**Methods**

A national online cross-sectional survey was conducted among 736 Australian residents aged 18 years and over. To assess knowledge and attitudes regarding crystal methamphetamine, participants were asked to complete several validated scales. Those who had not used CITI prior to the study, were asked to interact with the site and complete a follow-up survey a month later, which included repeated baseline measures.

**Results**

Preliminary findings indicate that Cracks in the Ice is perceived as helpful, strong in its evidence base and non-stigmatising. 64% of participants who engaged with the site before and after the redesign reported that the new design improved the site. Preliminary results suggest that exposure to the site may be associated with an improvement in knowledge and a decrease in stigmatising attitudes. Interaction with CITI also lead to a higher likelihood of participants seeking help for either their methamphetamine use, or a friend or family members use.

**Conclusions**

The results of this study indicate that CITI is achieving its aim of disseminating evidence-based and useful resources to the community and may improve knowledge and reduce stigma.

### O11 Identification of barriers and application of a theoretical framework to develop strategies supporting sustainment of a physical activity intervention in Australian primary schools

#### Adam Shoesmith^1^, Alix Hall^1^, Luke Wolfenden^2^, Rachel C Shelton^3^, Cassandra Lane^1^, Nicole McCarthy^1^, Edward Riley-Gibson^1^, Nicole Nathan^2^

##### ^1^School of Medicine and Public Health, Faculty of Health and Medicine, University of Newcastle, NSW, Australia; ^2^Hunter New England Population Health, Hunter New England Local Health District, NSW, Australia; ^3^Department of Sociomedical Sciences, Mailman School of Public Health, Columbia University, NY, USA

*Implementation Science 2023,* **18(2):**O11

**Background**

This study describes factors influencing sustainment of a school physical activity (PA) program and the application of theoretical frameworks to guide development of an intervention to sustain its delivery.

**Methods**

A multi-strategy intervention was co-developed with health and education policy makers and practitioners using the following steps: (1) Identification of sustainment determinants via: i) systematic reviews; ii) surveys with classroom teachers; and iii) interviews with school staff (2) Identification of potential sustainment strategies: barriers were organised according to the Integrated Sustainability Framework. Potential sustainment strategies were identified through surveys with 200 teachers. Theoretical mapping was used to link possible strategies to key barriers (3) Strategies were reviewed by key stakeholders to ensure their feasibility and acceptability. Final strategies were described according to a sustainment-explicit glossary.

**Results**

Key barriers to program sustainment were lack of organisational leadership and support, organisational readiness and resources, staff turnover, perceived policy alignment and workplace socio-cultural factors. Strategies perceived most useful by teachers to support sustainment were the provision of PA equipment packs (85%), a handover package to upskill new staff (78%), and delivery of professional learning modules (78%). Following theoretical mapping, a multi-component intervention was developed, including: (i) centralized support; (ii) reminders; (iii) principal mandates; (iv) sharing local knowledge; (v) building coalitions to share resources; (vi) distributing educational materials; and (vii) involving end-users.

**Conclusions**

This will be one of the first studies globally to test the effectiveness of a multi-component sustainability intervention to support sustainment of a school PA program.

### O12 Scaling up an effective m-health lunchbox program targeting parents in NSW primary schools

#### Katie Robertson^1^, Rachel Sutherland^1,2,3^, Courtney Barnes^2,3^, Lisa Janssen^2^, Aimee Mitchell^2,3^, Jannah Jones^2,3^, Alison Brown^2,3^, Daniel Groombridge^1^, Nicole Nathan^2,3^, Luke Wolfenden^1,2,3^

##### ^1^Hunter New England Population Health, Hunter New England Local Health District, NSW, Australia; ^2^School of Medicine and Public Health, Faculty of Health and Medicine, University of Newcastle, NSW, Australia; ^3^National Centre of Implementation Science, University of Newcastle, NSW, Australia

*Implementation Science 2023,* **18(2):**O12

**Background**

Over 85% of Australian primary school students bring a packed lunch to school containing more than 3 serves of discretionary food items, impacting on health and educational outcomes. We report on the development, pilot, optimisation and scale-up of the SWAP IT lunchbox program.

**Methods**

A seven-step program of work has been undertaken including: 1) formative research to identify parental barriers and behaviour change techniques (BCT) that underpinned the m-health intervention; 2) pilot RCT to evaluate acceptability, feasibility and potential efficacy; 3) intervention optimisation study prior to the fully powered trial; 4) hybrid effectiveness-implementation trial; 5) dose trial to identify core components and maximise cost-effectiveness; 6) evaluation of dissemination methods; and 7) scale-up.

**Results**

The SWAP IT program was developed to address five common parental barriers to packing healthy lunchboxes: time, cost, convenience, child preference and knowledge. BCT’s were incorporated into the intervention delivered via an existing school communication app, which was acceptable to 95% of parents during the pilot. Optimised lunchbox messages were then incorporated into the hybrid implementation-effectiveness trial in 36 schools across NSW which demonstrated a decrease in mean energy (kJ) content of discretionary foods packed in lunchboxes (-117.26kJ; P<0.01; n=3022). Results from the dose trial demonstrated no significant change in the effectiveness of SWAP IT when program costs decreased from $6.02 to $0.07 per student, making SWAP IT appropriate for large-scale dissemination.

**Conclusions**

This scalable m-health intervention has the potential to improve the health of populations by enhancing the nutritional quality of school lunchboxes.

### O13 A pilot randomised controlled trial to increase the sustainment of an indoor-outdoor free play program in early childhood education and care services following the introduction of outdoor free play guidelines

#### Noor Imad^1^, Nicole Pearson^2^, Alix Hall^3^, Adam Shoesmith^3^, Nicole Nathan^3^, Luke Giles^2^, Alice Grady^3^, Serene Yoong^4^

##### ^1^School of Health Sciences, Department of Nursing and Allied Health, Swinburne University of Technology, Hawthorn, VIC, Australia; ^2^Hunter New England Population Health, Hunter New England Local Health District, NSW, Australia; ^3^School of Medicine and Public Health, Faculty of Health and Medicine, University of Newcastle, NSW, Australia; ^4^Global Centre for Preventive Health and Nutrition, Institute for Health Transformation, Deakin University, VIC, Australia

*Implementation Science 2023,* **18(2):**O13

https://doi.org/10.3390/ijerph20065043

### O14 Cross-sectional study describing factors of sustainment of physical activity and nutrition interventions in childcare services

#### Noor Imad^1^, Alix Hall^2^, Nicole Nathan^2^, Adam Shoesmith^2^, Nicole Pearson^3^, Melanie Lum^3^, Serene Yoong^4^

##### ^1^School of Health Sciences, Department of Nursing and Allied Health, Swinburne University of Technology, Hawthorn, VIC, Australia; ^2^School of Medicine and Public Health, Faculty of Health and Medicine, University of Newcastle, NSW, Australia; ^3^Hunter New England Population Health, Hunter New England Local Health District, NSW, Australia; ^4^Global Centre for Preventive Health and Nutrition, Institute for Health Transformation, Deakin University, VIC, Australia

*Implementation Science 2023,* **18(2):**O14

**Background**

Many evidence-based physical activity and nutrition interventions exist within the childcare setting, however, they are not implemented in an ongoing way and thus are poorly sustained. There is a need to identify potential factors that may impact the sustainment of these interventions. The aim of this study is to identify and describe the factors related to the sustainment of physical activity and nutrition intervention in childcare services.

**Methods**

A cross-sectional study was undertaken with a nationally representative sample of 400 childcare services. Factors related to the sustainment of physical activity and nutrition interventions were assessed using a 29-item measure reflecting four domains of the Integrated Sustainability Framework (Outer Contextual Factors, Inner Contextual Factors, Processes and Characteristics of the Intervention) for interventions that supervisors reported as currently implementing. Participants responded using a 5-point Likert scale, with responses ranging from 1 (strongly disagree) to 5 (strongly agree). Domain scores were calculated for each service by averaging item responses.

**Results**

Preliminary data from 407 Australian childcare services nationally found that the domains; Processes (mean = 3.8), which includes factors of partnership/engagement and training/support/supervision and Outer Contextual Factors (mean = 3.8), which includes factors of policy and legislation, and socio-political context had the lowest mean scores.

**Conclusions**

This study suggests that factors related to the Processes and Outer Contextual Factors domains may need to be considered when developing an intervention to increase the sustainability of implementing physical activity and nutrition interventions in childcare settings.

### O15 Exploring the impact of a dissemination strategy on family day care educators’ adoption of outdoor free play guidelines introduced in response to COVID-19: A randomised controlled trial

#### Melanie Lum^1^, Sze Lin Yoong^2^, Kathryn Reilly^1,3^, Jacklyn Jackson^1^, Heidi Turon^1^, Luke Wolfenden^1^, Alice Grady^4^

##### ^1^School of Medicine and Public Health, Faculty of Health and Medicine, University of Newcastle, NSW, Australia; ^2^Centre for Preventive Health and Nutrition, Institute for Health Transformation, Deakin University, VIC, Australia; ^3^Hunter Medical Research Institute, NSW, Australia; ^4^Hunter New England Population Health, Hunter New England Local Health District, NSW, Australia.

*Implementation Science 2023,* **18(2):**O15

https://doi.org/10.1093/her/cyad014

## Successfully implementing novel practices in healthcare

### O16 Implementing genomics into practice within nongenetic paediatric settings using implementation science and transdisciplinary co-production

#### Hossai Gul^1^, Stephanie Best^2^, Janet Long^3^, Ellenore Martin^4^, Lucinda Murray^5^, Vanessa Fitzgerald^6^, Frances Rapport^3^, Mike Field^5^, Jeffrey Braithwaite^3^

##### ^1^SPHERE, TD School, University of Technology, Sydney, NSW, Australia; ^2^Peter MacCallum Cancer Centre, Melbourne, VIC, Australia; ^3^Australian Institute of Health Innovation, Macquarie University, Sydney, NSW, Australia; ^4^Australian Genomics, VIC, Australia; ^5^Hunter New England Local Health District, NSW, Australia; ^6^NSW Ministry of Health, NSW, Australia

*Implementation Science 2023,* **18(2):**O16

**Background**

Genomic mainstreaming is the implementation of genomic testing as routine practice within nongenetic settings. The aim of this body of work was to conduct a holistic implementation needs assessment that would guide the development of evidence-informed implementation strategies in support of real-world genomic mainstreaming efforts within nongenetic paediatric settings via an integrated model of care involving clinical genetics services.

**Methods**

A sequence of three studies were conducted via a mixed method methodology and structured by the process model Implementation Mapping (IM) to guide the development of implementation strategies. The research approach was informed by a complex adaptive systems lens and guided by transdisciplinary co-production.

**Results**

Study one began with an implementation needs assessment within genetics services via qualitative semi-structured interviews (n=14 participants, clinical genetics professionals), with resultant data analysed using (1) the Interactive Systems Framework (ISF) for mapping the implementation system, (2) pathway mapping techniques to visualise changes required in processes and practices, and (3) the Consolidated Framework for Implementation Research (CFIR) to assess the barriers and facilitators to implementation within genetic services. In study two an implementation needs assessment was conducted within paediatric services via a cross-sectional survey (n=114 respondents, paediatricians) analysed using descriptive statistics and semi-structured interviews (n=22) analysed using the TDF framework.

**Conclusions**

In study three, using a combination of five implementation science tools, the findings from studies one and two were integrated to develop forty evidence-informed, discrete implementation strategies with specifications ready to be used by groups across health systems involved in real-world implementation efforts.

### O17 Developing an implementation strategy for routine collection of patient-reported outcome measures (PROMS) in multidisciplinary teams

#### Laura Jolliffe, Nadine Andrew, Velandai Srikanth, Richard Beare, Kate Noeske, David Snowdon

##### Peninsula Health, VIC, Australia

*Implementation Science 2023,* **18(2):**O17

**Background**

Little is known about implementing patient reported outcome measures (PROMs) in multidisciplinary settings. To date, developing implementation approaches for PROMs have largely focused on single-disciplines with an assumption that clinicians can forecast potential implementation barriers and enablers. Our study aimed to develop a large-scale theory-informed implementation approach after 12 months of routine PROM collection.

**Methods**

Semi-structured interviews were completed with allied health clinicians who were involved in the routine collection of PROMs in a multidisciplinary community rehabilitation program (across three sites of a large public health service). Data from interviews were independently coded by two authors, and mapped against the Theoretical Domains Framework (TDF) and the Behaviour Change Wheel (BCW). We applied the APEASE (Acceptability, Practicability, Effectiveness, Affordability, Side-effects, and Equity) criteria and used a consensus approach to refine the multifaceted strategies.

**Results**

21 interviews were conducted, and four themes emerged: 1) The Impact of PROMS on patient centred-care; 2) Considerations for validity of PROMS; 3) Service-level impact of embedding PROMS; and, 4) Practical issues of embedding PROMS within the service. These mapped against seven TDF domains, and five hypothetical strategies were developed. Key behaviour change techniques underpinning the strategies include: restructuring the physical environment, incentivisation, persuasion and education, enablement, and, social support.

**Conclusions**

The implementation approach highlights the importance of automating processes, engaging with site champions, routinely reporting PROM data to clinical teams, and empowering clinicians to use this data to inform service provision.

### O18 Development of an implementation-focused logic model framework: a practical example of use to design and support complex care provision for children with a hard-to-treat-cancer

#### Skye McKay^1^, Carolyn Mazariego^1^, Mark Dobson^2^, Elijah Tyedmers^1^, Lauren Kelada^3,4^, Brittany McGill^3,4^, Rebecca Daly^3,4^, Claire E. Wakefield^4,5^, David Ziegler^2,5^, Natalie Taylor^1^

##### ^1^School of Population Health, UNSW Medicine and Health, UNSW Sydney, NSW, Australia; ^2^Kids Cancer Centre, Sydney Children’s Hospital, NSW, Australia; ^3^Discipline of Pediatrics and Child Health, School of Clinical Medicine, UNSW Medicine & Health, UNSW Sydney; ^4^Behavioural Sciences Unit, Kids Cancer Centre, Sydney Children’s Hospital, Randwick, NSW, Australia; ^5^School of Clinical Medicine, UNSW Medicine and Health, UNSW Sydney, NSW, Australia

*Implementation Science 2023,* **18(2):**O18

**Background**

Usage of implementation research logic models (IRLMs) enable better transparency of implementation science research and intended outcomes. Complex clinical interventions can benefit from novel resources to support implementation, with some requiring a granular level of strategy development. Existing IRLMs do not capture all three layers, making it challenging to delineate between influencing factors and mechanistic effects. Here we demonstrate a novel IRLM designed for ProCure, a database to streamline the novel medicines access pathways for paediatric oncology healthcare professionals (HCPs)

**Methods**

The implementation science team used a consensus-based approach to amend a Smith, JD et al (2020) IRLM template to demonstrate inter-relationships between the clinical intervention, implementation intervention, and implementation strategies. Analysis of 17 HCP interviews enabled coding of contextual barriers and facilitators (i.e., determinants) to the Consolidated Framework for Implementation Research (CFIR).

**Results**

Precision medicine is the IRLM ‘Clinical Intervention’, directly impacting patient outcomes, implementation context, and ProCure design. As the ‘Implementation Intervention’, ProCure aims to facilitate the medicines access pathway in precision medicine. Factors influencing ProCure implementation are stratified as CFIR coded determinants and targeted by Expert Recommendations for Implementing Change (ERIC) implementation strategies. Relationships between hypothesised mechanism of action and intended outcome(s) are represented using superscripts.

**Conclusions**

This IRLM provides a tailored theoretical model to capture the complexity between a clinical intervention, an implementation intervention, and deployed implementation strategies, using ProCure as an example. The causal relationships will be tested and refined throughout the study and measured with meaningful outcomes to enhance transparency, reproducibility and scale-up.

### O19 Process evaluation exploring implementation outcomes and barriers and facilitators of a Healthy Lifestyle for low back pain Program (HeLP) Intervention embedded in clinical care

#### Emma Robson^1,2^, Cassandra Lane^2^, Steven Kamper^3^, Jenna Hollis^2^, Simon Davidson^2^, Christopher Williams^3^, Priscilla viana da Silva^2^, Connor Gleadhill^2^, Rebecca Hodder^2^

##### ^1^Hunter New England Population Health, Hunter New England Local Health District, NSW, Australia; ^2^University of Newcastle, NSW, Australia; ^3^University of Sydney, NSW, Australia

*Implementation Science 2023,* **18(2):**O19

**Background**

We describe implementation outcomes of 1) fidelity; 2) adoption; 3) acceptability, appropriateness and feasibility; and 4) barriers and facilitators of engagement and delivery of a Healthy Lifestyle Program (HeLP) for low back pain.

**Methods**

The RCT included 346 adults with chronic low back pain and at least one health risk factor randomised to HeLP or guideline care. HeLP included: consultations, resources, and referral to telephone services for lifestyle risk factors, over 26 weeks. We used a sequential mixed methods design to evaluate HeLP participant and clinician data. We collected quantitative data via fidelity checklists, administrative records, and surveys. We collected qualitative data via semi-structured interviews and focus groups with participants and clinicians. We used descriptive statistics to analyse quantitative data, thematic analyses for qualitative data, and triangulation to integrate data and identify meta-themes.

**Results**

Intervention fidelity was high (>90% delivered). Participants attended a mean 3.2 consultations, 54% engaged with telephone services and 26% used online resources. Clinicians and participants found the intervention acceptable and addressing lifestyle factors was considered appropriate for back pain management. Perceptions varied on the acceptability and appropriateness of telephone services, and whether parts of the program met individual patient needs. Clinicians’ delivery barriers included low self-efficacy in behaviour change communication skills, while enablers were behaviour change communication skills training and practice observation and feedback.

**Conclusions**

Participants and clinicians appeared satisfied with HeLP, however adoption of discrete components varied. Adaptations to improve clinicians’ behaviour change communication skills and individualisation of care may optimise future implementation.

### O20 A clinical implementation trial to inform successful genomic medicine strategies in practice: improving tumour testing and genetic services referral for Lynch syndrome at 7 major hospitals in Australia

#### Julia Steinberg^1^, Priscilla Chan^2^, Sarsha Yap^1^, April Morrow^2^, Gabriella Tiernan^2^, Yoon-Jung Kang^1^, Emily He^1^, Rhiannon Edge ^1^, Deborah Debono^3^, Bonny Parkinson^4^, Hide and Seek Clinical Project Team^1^, Karen Canfell^1^, Finlay Macrae^5^, Kathy Tucker^2,6^, Emily Hogden^2^, Natalie Taylor^2^

##### ^1^The Daffodil Centre, University of Sydney, NSW, Australia; ^2^University of NSW, NSW, Australia; ^3^University of Technology, Sydney, NSW, Australia; ^4^Macquarie University, NSW, Australia; ^5^The Royal Melbourne Hospital, Melbourne, VIC, Australia; Prince of Wales Hospital, Sydney, NSW, Australia

*Implementation Science 2023,* **18(2):**O20

**Background**

To inform implementation of effective genomic medicine, current gaps and successful strategies to support clinician practice change for well-established applications can provide important insights. Lynch syndrome (LS) causes 3-5% of colorectal cancers (CRCs), with long-standing guidelines recommending LS tumour testing of all CRC patients, but substantial heterogeneity in practice. Our trial compared the effectiveness of two structured implementation approaches (theory-based/non-theory-based) to improve risk-appropriate LS tumour testing and referral to genetics services.

**Methods**

Seven major Australian hospitals were randomly allocated to two trial arms. Hospital and genetics services data for 01/01/2017-31/12/2018 were used to identify hospital-specific practice gaps (total n=1,624 CRC patients). At each hospital, a health service professional was trained in evidence-based implementation to form stakeholder teams to identify target behaviours for change and associated barriers (using process mapping, questionnaires, focus groups), then co-design and implement targeted strategies. Trial arms differed only in the use of theory to identify barriers and design strategies.

**Results**

Pre-trial, risk-appropriate LS tumour testing and referral was complete 2 months post-resection for 76.5% and 74.9% of patients in theory-based and non-theory-based arms, respectively (aRR=1.02, 95%CI 0.74-1.41). Clinical practice differed in six key areas, including multidisciplinary input and application of testing guidelines. With implementation of site-specific strategies, risk-appropriate tumour testing and referral 2 months post-resection increased to 89.1% of patients in the theory-based arm but decreased to 65.9% in the non-theory arm (aRR 1.31, 95%CI 1.16-1.47). Hospital-level changes were variable and likely affected by COVID-19.

**Conclusions**

Findings suggest theory-based implementation science approaches might support successful integration of genomics into clinical care.

### O21 Hey Vocera, call the doc: Evaluation of the Vocera Badge communication device

#### Jacky Hanh^1^, Tony Tu^1^, Olivia King^1^, Shona Hanson^1^, Adeola Bamgboje-Ayodele^2^, Melissa Baysari^2^, Aaron Jones^1^

##### ^1^Sydney Local Health District, Sydney, NSW, Australia; ^2^University of Sydney, Sydney, NSW, Australia

*Implementation Science 2023,* **18(2):**O21

**Background**

Increased COVID-19 hospital admissions with greater clinical complexity during the Delta wave resulted in changes to ward layouts and reliance on isolation rooms. Maintaining infection control practices introduced challenges to existing communication methods and created complex barriers to effective patient care. The Vocera Badge was identified as a potential solution because it enables portable hands-free voice-activated communication to other staff, landlines and mobiles, while being worn underneath personal protective equipment. This project aimed to evaluate whether Vocera improved staff communication efficiency, user perceptions of the device, and device implementation strategies at a tertiary metropolitan hospital.

**Methods**

Vocera was implemented for medical, nursing and clerical staff within the emergency department. The facility Bed Manager and After-hours Operational Nurse Manager were also strategically included. Scenario-based usability testing was performed to measure communication efficiencies gained. User perceptions of Vocera and its implementation were evaluated with surveys, focus groups and usage data analyses.

**Results**

Preliminary results from usability testing found reductions in communication delay times from 70 seconds to 18 seconds. Communications with delays of less than 15 seconds improved from 0% to 46%. This equated to time savings of 92 hours over a 4-week period. Analyses of the surveys, focus groups and usage data are currently in progress.

**Conclusions**

This project demonstrated significant increases in communication efficiency with Vocera. Planned analyses of surveys, focus groups and Vocera usage data will elucidate effective implementation strategies. These findings will guide large-scale implementation across the health district and more broadly across NSW and Australia.

### O22 Implementing improvements for complex rare conditions: the neurofibromatosis networked model of care in NSW

#### Hossai Gul^1^, Sue-Faye Siow^2^, Jane Fleming^2^, Suzana Milosjavic^2^, Martin Good^2^, Manoj Menezes^3^, Tina Gonzalez^2^, Sally Maspero^2^, Kristi Jones^3^, Yemima Berman^2^

##### ^1^ SPHERE, TD School, University of Technology, Sydney, NSW, Australia; ^2^North Sydney Local Health District, NSW, Australia; ^3^Westmead Children’s Hospital, Sydney, NSW, Australia

*Implementation Science 2023,* **18(2):**O22

**Background**

The neurofibromatoses are a group of genetic conditions associated with multisystemic complications resulting in complex care needs. Delivery of care is fragmented due to local health district boundaries, and subspecialist services are unable to be duplicated due to the highly specialised nature of treatments. The aim of this study was to identify and implement the model of care that would provide neurofibromatosis patients in NSW the highest quality care.

**Methods**

A sequential mixed method of survey and semi-structured interviews were conducted with identified health professionals delivering adult and/or paediatric services for patients with NF1, NF2 and schwannomatosis. Survey results were analysed using descriptive statistics and qualitative data was analysed using thematic analysis.

**Results**

Surveys were completed by 47 participants, representing a diverse cohort of health professionals from specialist physicians, specialist surgeons, to allied health professionals covering services across 17 local health districts within NSW. Of these 21 opted-in for semi-structured interviews. The findings showed a pre-existing networked model of care whereby a variety of health professionals conversant with neurofibromatosis deliver subspecialist services across the lifetime and across the different types of neurofibromatoses with two concentrations of services (1 adult and 1 children) within the state. The main areas in need of improvement were identified: transition from paediatric care to adult care, a need for a centralised platform for resources and services, and better referral pathways/protocols. Interventions were put in place across the model of care and data is being collected on effectiveness and implementation in parallel.

**Conclusions**

Improvements within rare complex models of care requires mapping of the system and intervening within key leverage points to develop and implement evidence-informed interventions on a continuous basis.

### O23 Lung cancer screening in Australia: using implementation science frameworks to accelerate translation

#### Nicole Rankin^1^, Rachael H. Dodd^2,3^, Kate L.A. Dunlop^2,3^, Henry M. Marshall^4,5^, Joel Rhee^6^, Mei Ling Yap^7^, Sue McCullough^6^, Sarah York^8^, Emily Stone^6,9^

##### ^1^University of Melbourne, VIC, Australia; ^2^ Daffodil Centre, University of Sydney and Cancer Council NSW, NSW, Australia; ^3^ University of Sydney, NSW, Australia; ^4^The University of Queensland, QLD, Australia; ^5^The Prince Charles Hospital, QLD, Australia; ^6^The University of New South Wales, NSW, Australia; ^7^Collaboration for Cancer Outcomes, Research and Evaluation, Ingham Institute, The University of New South Wales, NSW, Australia; ^8^Sydney School of Public Health, the University of Sydney, Sydney, NSW, Australia; St Vincent's Hospital, Sydney, NSW, Australia

*Implementation Science 2023,* **18(2):**O23

**Background**

Lung cancer screening in high-risk populations, using low dose computed tomography has the potential to save thousands of lives by detecting early-stage curable disease. Significant progress towards implementation includes a recommendation that the Australian Government fund a national program, feasibility trials, government-commissioned scoping work and implementation research. This presentation aims to describe how implementation science frameworks are being utilised in generating pre-implementation evidence.

**Methods**

We conducted two qualitative studies about lung cancer screening acceptability and feasibility. Study 1) semi-structured telephone interviews were completed with 39 individuals from the International Lung Screening Trial who had participated or declined to screen; we used the COM-B model of behaviour change for analysis. Study 2) we conducted 24 focus groups with 84 key stakeholders (e.g., healthcare professionals); we used the Consolidated Framework for Implementation Research for analysis. The CFIR-ERIC matching tool was used to identify potential implementation strategies.

**Results**

The COM-B framework showed that motivation alone is insufficient to engage high-risk participants in screening; opportunity and capability must inform selection of implementation strategies that target individual behaviour change. The focus groups analysis elicited determinants that were mapped to CFIR constructs, of which “readiness for implementation”, “planning” and “executing” were most relevant. The matching tool identified at least 12 potential implementation strategies at health system, provider, participant and policy levels that should be considered.

**Conclusions**

Implementation science frameworks should be utilised in pre-implementation research. The findings provide an evidence-based foundation for selecting and designing implementation strategies for a national lung cancer screening program.

### O24 Using process mapping to capture variability within paediatric cancer survivorship services: understanding factors influencing implementation of the Engage program

#### Skye McKay^1^, Carolyn Mazariego^1^, Joseph Elias^1^, Jordana McLoone^2,3^, Christina Signorelli^2,3^, Claire E Wakefield^2,3^, Richard Cohn^2,3^, Natalie Taylor^1^

##### ^1^School of Population Health, UNSW Medicine and Health, UNSW Sydney, NSW, Australia; ^2^Behavioural Sciences Unit, Kids Cancer Centre, Sydney Children's Hospital, Randwick, NSW, Australia; ^3^School of Clinical Medicine, UNSW Medicine & Health, Discipline of Paediatrics, UNSW Sydney, NSW, Australia

*Implementation Science 2023,* **18(2):**O24

**Background**

Process mapping can provide an in-depth understanding of clinical processes, necessary for successful implementation of evidence-based clinical interventions. The Engage program innovates a distance-delivered multi-disciplinary model of survivorship care. Process mapping was applied at three hospital sites to understand existing survivorship care delivery and identify factors influencing implementation of Engage at each stage of the care pathway.

**Methods**

Survivorship-service healthcare professionals (HCPs) were invited to participate in semi-structured interviews guided by the Consolidated Framework for Implementation Research (CFIR). A draft process map facilitated step-by-step discussion of the clinical practices involved from the time a patient completes treatment to commences survivorship care. Interviews explored how core components of Engage could be integrated into existing practices.

**Results**

Data from 16 HCPs interviews produced three distinct survivorship service process maps and identified unique and shared factors impacting implementation of Engage. Common barriers include: patient identification; obtaining GP referrals; scheduling multidisciplinary team (MDT) meetings and compiling the MDT letter. However, underlying contextual causes for these shared barriers often differed between sites, requiring implementation strategies to be tailored to fit site-specific needs. Key facilitators to implementation included an experienced survivorship care team and established telehealth services.

**Conclusions**

Process mapping successfully identifies site-specific variation in complex clinical processes and facilitates a shared understanding of the current systems and factors that affect implementation. This deeper knowledge allows for the development of a suite of implementation strategies, which can be adapted to support implementation and scale-up at additional sites.

### O25 Capturing variability in the Lynch syndrome genetic referral pathway

#### April Morrow^1^, Julia Steinberg^2^, Priscilla Chan^1^, Gabriella Tiernan^1^, Elizabeth Kennedy^2^, Natasha Erogoff^3^, Desiree Hilton^4^, Lucien Sankey^5^, Rebecca Venchiarutti^2^, Anne Hayward^7^, Amy Pearne^2^, Skye McKay^1^, Deborah Debono^8^, Emily Hogden^1^, Natalie Taylor^1^

##### ^1^University of NSW, Sydney, NSW, Australia; ^2^Daffodil Centre, University of Sydney, NSW, Australia; ^3^The University of Newcastle, NSW, Australia; ^4^Westmead Hospital, NSW, Australia; ^5^Monash Health, VIC, Australia; ^6^The University of Sydney, Sydney, NSW, Australia; ^7^Royal Melbourne Hospital, VIC, Australia; ^8^University of Technology, NSW, Australia

*Implementation Science 2023,* **18(2):**O25

https://doi.org/10.1093/tbm/ibad009

### O26 How to effectively implement preoperative anaemia and iron deficiency screening, evaluation and management pathways using the CFIR-ERIC approach

#### Alana Delaforce^1^, Shannon Farmer^2^, Jed Duff ^3^, Judy Munday^3^, Kristin Miller^4^, Lynne Glover^4^, Chris Corney^4^, Gareth Ansell^4^, Naadir Gutta^4^, Haitham Tuffaha^5^, Janet Hardy^4^, Cameron Hurst^6^

##### ^1^Commonwealth Scientific and Industrial Research Organisation (CSIRO), ACT, Australia; ^2^Department of Haematology, Royal Perth Hospital, WA, Australia; ^3^Centre for Healthcare Transformation/School of Nursing, Queensland University of Technology, QLD, Australia; ^4^Mater Health Services, QLD, Australia; ^5^Centre for the Business and Economics of Health, University of Queensland, QLD, Australia; ^6^Charles Sturt University, NSW, Australia

*Implementation Science 2023,* **18(2):**O26

https://doi.org/10.1111/trf.17287

### O27 Impact of core versus enhanced implementation strategies on adherence to a clinical pathway for managing anxiety and depression in cancer patients in routine care: A cluster randomised controlled trial

#### Heather L Sheperd^1,2^, Phyllis Butow^2^, Mona Faris^2^, Joanne Shaw^2^, Patrick Kelly^3^, Marnie Harris^2^, Jessica Cuddy^2^, Lindy Masya^2^, Liesbeth Geerligs^2^, Brian Kelly^4^, Afaf Girgis^5^, Nicole Rankin^6^, Philip Beale^7^, Thomas Hack^8^, Laura Kirsten^9^, Haryana Dhillon^2^, Peter Grimison^10^, Rosalie Viney^11^, Josephine Clayton^12^, Tim Schlub^2^, and the ADAPT Program Group

##### ^1^Susan Wakil School of Nursing and Midwifery, Faculty of Medicine and Health, The University of Sydney, Sydney, NSW, Australia; ^2^School of Psychology, Psycho-Oncology Co-operative Research Group, The University of Sydney, Sydney, NSW, Australia; ^3^Sydney School of Public Health, Faculty of Medicine and Health, The University of Sydney, Sydney, NSW, Australia; ^4^School of Medicine and Public Health, University of Newcastle, NSW, Australia; ^5^South West Sydney Clinical Campuses, UNSW Medicine & Health, University of New South Wales, Kensington, NSW, Australia; ^6^Centre for Health Policy, Faculty of Medicine, Dentistry and Health Sciences, Melbourne, NSW, Australia; ^7^Concord Hospital, Sydney Local Health District, NSW, Australia; ^8^College of Nursing, Rady Faculty of Health Sciences, University of Manitoba, Winnipeg, Manitoba, Canada; ^9^Nepean Blue Mountains Local Health District, NSW, Australia; ^10^Chris O’Brien Lifehouse, Camperdown, NSW, Australia; ^11^Centre for Health Economics Research and Evaluation, University of Technology, Sydney, NSW Australia; ^12^The Palliative Centre, Greenwich Hospital, NSW, Australia

*Implementation Science 2023,* **18(2):**O27

**Background**

Optimal strategies to facilitate implementation of evidence-based clinical pathways are unclear. We evaluated a core and an enhanced implementation strategy to facilitate implementation of a clinical pathway for the management of anxiety and depression (A/D) in patients with cancer (ADAPT CP).

**Methods**

Twelve cancer services were cluster randomised to a Core versus Enhanced implementation strategy for 12 months. Core strategy included a Lead Team with champions, awareness campaigns, staff training, feedback reports, telephone/online support. Enhanced strategy added monthly meetings, proactive advice, tailored awareness campaigns. Patients were introduced to the ADAPT CP as routine care, completed screening, and allocated an A/D step of 1-5 (minimal/mild/moderate/severe/very severe), with referral for additional support. Multi-level mixed-effect regression analyses examined impact of implementation strategy on ADAPT CP adherence (binary primary outcome: adherent: ≥70% ADAPT CP components achieved; or non-adherent: <70% achieved), continuous adherence was a secondary outcome. Interaction between implementation strategies and A/D step severity was explored.

**Results**

Of 1,280 registered patients, 696 (54%) completed screening, 1,323 screening events in total (883 Core, 440 Enhanced). The main effect of implementation strategy on adherence was non-significant in binary and continuous analyses, however, A/D step was significant. Adherence was higher for Step 2 than other steps (p=0.001). Interaction between implementation strategy and A/D step was significant (p=0.02) in the continuous adherence analysis: adherence with the enhanced strategy was significantly higher (7.5%points) for Step 3 (p=0.048) and trending to significance for Step 4.

**Conclusions**

Results support ongoing implementation effort for the first year of implementation to ensure uptake and sustainment of clinical pathways in over-burdened clinical services.

### O28 How actionable are infection prevention and control guidelines in residential aged care? A document analysis based on a behaviour specification framework

#### Joanne Tropea^1,2^, Jill Francis^3^, Lyn-li Lim^4^, Noleen Bennett^4^, Kwang Lim^1,2^, Kirsty Buising^1,2^, Deirdre Fetherstonhaugh^5^, Sanne Peters^3^

##### ^1^Royal Melbourne Hospital, Melbourne, VIC, Australia; ^2^Department of Medicine, Royal Melbourne Hospital, University of Melbourne, VIC, Australia; ^3^School of Health Sciences, University of Melbourne, Melbourne, VIC, Australia; ^4^VICNISS, Peter Doherty Institute, University of Melbourne, VIC, Australia; ^5^Australian Centre for Evidence Based Aged Care, La Trobe University, VIC, Australia

*Implementation Science 2023,* **18(2):**O28

**Background**

Older people living in residential aged care are susceptible to transmissible infections such as influenza, COVID-19, and gastroenteritis. Effective infection prevention and control (IPC) practice in residential aged care is therefore imperative. To enable this, national and aged care provider-level IPC guidelines need to be specific enough to be actionable by residential aged care staff and organisations. The aim of this study was to assess the actionability of IPC national guidelines and residential aged care policies and procedures. We chose to examine the guidelines around healthcare associated infection (HAI) surveillance in residential aged care.

**Methods**

A content analysis of the Australian IPC guidelines, and IPC policies and procedures from Victorian residential aged care facilities was conducted. Data extraction, coding and interpretation of findings were directed by the action-actor-context-target-time (AACTT) framework.

**Results**

National guidelines did not specify recommendations related to HAI surveillance but include general statements of support for data collection on HAI and outbreaks, suggest best epidemiologic principles that should be applied in data collection, and suggest that data should be fed back to appropriate staff groups and administrators. Provider-level policies and procedures varied in specificity.

**Conclusions**

While it is recommended that aged care providers undertake HAI surveillance, national guideline recommendations are open to interpretation and are not specific or actionable. Provider-level guidelines also need improving to facilitate actionability. To increase uptake of effective HAI surveillance in residential aged care, local policies and procedures need to be written with greater behavioural specificity.

### O29 The expected value of implementation: The use of iterative expert elicitation and scenario analyses within decision-analytic models of health services

#### Andrew Partington^1,2^, Jonathan Karnon^1^

##### ^1^Flinders Health and Medical Research Institute, Flinders University, SA, Australia; ^2^Australian Institute of Health Innovation, Macquarie University, NSW, Australia

*Implementation Science 2023,* **18(2):**O29

**Background**

Health economists can inform the management of integrated services, rather than merely the adoption of discrete and disjointed products e.g., pharmaceuticals and devices. But this is not so simple. During ex-ante value-based planning of interventions, consideration is required of implementation fidelity within complex and dynamic systems. Potential misalignments require adaptations to resourcing, but also our expectations of effects and, therefore, cost-effectiveness i.e., value and success.

**Methods**

While embedded within Local Health Networks in South Australia, we conducted interviews and retroductive analyses to conceptualise decision problems and the logic behind interventions. We then conducted expert elicitation of quantitative estimates of expected future effects, which were used to model the expected cost-effectiveness of theorised service interventions. Finally, scenario analyses were used to explore the implementation costs necessary to minimise the likelihood of poor fidelity and sustainability.

**Results**

Expected effects include non-inferior outcomes, financial savings, and repurposed capacity. There have been moderate levels of disagreement among stakeholders regarding the scale and uncertainty of expected effects ex-ante. Theorised adaptations included the need for unscheduled meetings, backup/redundant equipment, and “exceptional circumstance” responsibilities. Steep learning effects are also expected regarding the “risk appetite” of referrers. Modelled cost-effectiveness is sensitive to scenarios of poor adaptation and patient selection.

**Conclusions**

Modelled evaluations feed into business cases and living analytical models. While value is context-dependent, methods are generalisable. We highlight the potential of a Value of Implementation equation to price-in resources or “operational slack” for expected adaptations based on acceptable likelihoods of realised and sustained costs and effects.

### O30 Designing for implementation: co-design of a paediatric oncology medicines database (ProCure) to support complex care provision for children with a hard-to-treat cancer

#### Elijah Tyedmers^1^, Carolyn Mazariego^1^, Mark Dobson^2^, Skye McKay^1^, Lauren Kelada^2,3^, Brittany McGill^2,3^, Rebecca Daly^2,3^, Claire Wakefield^2^, David Ziegler^2,3^, Natalie Taylor^1^

##### ^1^School of Population Health, University of NSW Medicine and Health, Sydney, NSW, Australia; ^2^Kids Cancer Centre, Sydney Children’s Hospital, Randwick, NSW, Australia; ^3^School of Clinical Medicine, University of NSW Medicine and Health, University of NSW, Sydney, NSW, Australia

*Implementation Science 2023,* **18(2):**O30

**Background**

Co-design of technological interventions uses creative and participatory methods. In pediatric precision-medicine, treatment options identified by trials are often not approved and associated with ambiguous and time-consuming access pathways. This study aimed to use co-design methodologies to develop “ProCure”, a novel medicines access database that streamlines the application process for compassionate-use cancer therapies.

**Methods**

To promote ProCure’s implementation and ensure scalability, implementation science methodologies were used to guide the development and co-design process. Process mapping and implementation science frameworks were combined to explore healthcare professionals’ (HCP) perceived barriers and facilitators to current access pathways and their perceived acceptability to ProCure. HCPs participated in semi-structured interviews, guided by a process map depicting current novel medicine access pathways. Qualitative interview data were coded to the Consolidated Framework for Implementation Research (CFIR) to identify contextual barriers, explore perceived acceptability of ProCure and identify end-user needs.

**Results**

Key barriers to the current process were identified (e.g., resource-intensive applications to access medicines, time-sensitive decision-making, complicated pharmaceutical information), informing the co-design of ProCure. Most HCPs expressed perceived value in ProCure and intention to use it. Implementation strategies will be developed using the CFIR-ERIC (Expert Recommendations for Implementing Change) matching tool to guide implementation at the pilot site.

**Conclusions**

ProCure is perceived as an acceptable resource with potential to streamline off-label medicines access. End-user testing will use a mixed-methods approach to evaluate implementation determinants of ProCure. Combining process mapping and CFIR succeeded in informing ProCure’s co-design and readying the database for implementation and national scale-up.

## Sustaining change in health service processes and pathways

### O31 Implementation gaps in the use of clinical decision support systems for chronic obstructive pulmonary disease (COPD): A systematic review

#### Adeola Bamgboje-Ayodele^1^, David Borg^2^, Steven McPhail^2^, Melissa Baysari^1^

##### ^1^Biomedical Informatics and Digital Health, School of Medical Sciences, Charles Perkins Centre, Faculty of Medicine and Health, University of Sydney, NSW, Australia; ^2^Australian Centre for Health Services Innovation and Centre for Healthcare Transformation, School of Public Health and Social Work, Queensland University of Technology, Brisbane, Australia

*Implementation Science 2023,* **18(2):**O31

**Background**

Clinical decision support (CDS) systems have the potential to improve safety, quality and efficiency of care in various clinical domains including asthma, but the evidence regarding the use of CDS systems for patients with chronic obstructive pulmonary disease (COPD) in hospital or hospital-in-the-home settings has not yet been systematically reviewed. We aimed to describe existing COPD CDS systems and their impact on outcomes and identify barriers and facilitators to their implementation.

**Methods**

Databases (Medline, Embase, CINAHL, Scopus, Web of Science) were searched to identify relevant studies. Studies describing clinician-facing COPD CDS systems designed for, or implemented in, hospitals and hospital-in-the-home settings were included. A qualitative narrative synthesis was undertaken, guided by the RE-AIM framework (Reach, Effectiveness, Adoption, Implementation, Maintenance).

**Results**

Twelve studies reporting the use of CDS in hospital (n=7) and hospital-in-home (n=5) settings were included. Implementation efforts to REACH target users were scantly reported and low-to-medium ADOPTION rates were observed. The reported EFFECTIVENESS of the CDS systems was mixed. Only one study reported facilitators to the IMPLEMENTATION of CDS systems, none reported on barriers to the implementation of CDS systems and none reported any information on successful strategies to MAINTAIN implementation of CDS systems.

**Conclusions**

CDS systems offer enormous potential to facilitate clinical decision-making, but this is yet to be capitalised for the management of COPD. Many opportunities to optimise and evaluate the implementation and use of COPD CDS systems in hospital settings remain, including robust evaluation of their impact on patient, clinician, and health-service outcomes.

### O32 The implementation of best practice guidelines for incontinence-associated dermatitis in six hospitals in five Local Health Districts in New South Wales

#### Michelle barakat-Johnson^1,2,3^, Michelle Lai^1,2^, Shifa Basjarahil^4^, Jayne Campbell^5^, Michelle Cunich^1,2^, Gary Disher^6^, Samara Geering^7^, Natalie Ko^8^, Catherine Leahy^9^, Thomas Leong^1^, Eve McClure^1^, Melissa O'Grady^1^, Joan Walsh^4^, Kate White^1,2^, Fiona Coyer^10,11^

##### ^1^Sydney Local Health District, NSW, Australia; ^2^University of Sydney, NSW, Australia; ^3^Queensland University of Technology, QLD, Australia; ^4^ The Sutherland Hospital, NSW Australia; ^5^Hunter New England Local Health District, NSW, Australia; ^6^New South Wales Ministry of Health, NSW, Australia; ^7^South Western Sydney Local Health District, NSW, Australia; ^8^Concord Repatriation General Hospital, NSW, Australia; ^9^Western New South Wales Local Health District, NSW, Australia; ^10^University of Queensland, QLD, Australia; ^11^Royal Brisbane and Women's Hospital, QLD, Australia

*Implementation Science 2023,* **18(2):**O32

**Background**

Incontinence-associated dermatitis (IAD) is skin damage associated with exposure to urine/stool. In 2015, IAD best practice guidelines were published to improve prevention and management. From 2020, the IMBED study implemented these guidelines in New South Wales (NSW).

**Methods**

IMBED was conducted in six hospitals (four metropolitan, two regional/rural). At a macro level, the NSW Ministry of Health, Agency for Clinical Innovation, Clinical Excellence Commission, and Continence Foundation of Australia oversaw the design, provided access to new funding and promoted adaptability, implementation, and scalability of the intervention in the project’s course. At a meso level, each hospital developed an implementation strategy to enhance scalability and sustainability across their hospital, including planning for pre-implementation education, intervention adherence audits, post-implementation monitoring and reporting to the hospital’s skin integrity lead. Needs analyses were conducted at a micro level in each ward to understand readiness for change, barriers and facilitators to implementation which informed the hospital’s implementation strategy.

**Results**

Comparing pre- to post-implementation, hospital-acquired IAD prevalence lowered by 36.3%. Clinician knowledge of IAD aetiology and risk, classification and diagnosis, and prevention and management significantly improved. There was significant decrease in costs associated with the use of products not evidence-based to prevent or manage IAD, including creams such as zinc, and extra under-pads. Clinicians reported greater confidence with managing and preventing IAD.

**Conclusions**

IMBED has proven to be a successful exemplar of the adoption, implementation, scaling and sustaining of best practice guidelines to improve IAD prevention and management at a macro, meso and micro level.

### O33 Implementing a ward-based programme to improve care for older inpatients: process evaluation of the cluster randomised CHERISH trial

#### Alison Mudge^1,2^, Prue McRae^2^, Adrienne Young^2,3^, Irene Blackberry^4^, Karen Lee-Steere^2^, Sally Barrimore^5^, Tara Quirke^6^, Gillian Harvey^7^

##### ^1^University of Queensland Medical School, QLD, Australia; ^2^Royal Brisbane and Women's Hospital, QLD, Australia; ^3^Centre for Research in Geriatric Medicine, University of Queensland, QLD, Australia; ^4^John Richards Centre for Rural Ageing Research, Latrobe University, VIC, Australia; ^5^The Prince Charles Hospital, QLD, Australia; ^6^Dementia Training Australia, Australia; ^7^College of Nursing and Health Sciences, Flinders University, SA, Australia

*Implementation Science 2023,* **18(2):**O33

**Background**

Eat Walk Engage is a ward-based improvement programme using the i-PARIHS implementation framework to improve care of older people. It significantly reduced delirium in the four-hospital CHERISH cluster trial. The objective of this process evaluation was to understand how Eat Walk Engage worked across trial sites.

**Methods**

Prospective multi-method implementation evaluation on medical and surgical wards in four hospitals implementing Eat Walk Engage January 2016-May 2017. We assessed context, implementation (core components, implementation strategies and improvements) and mechanisms (practice changes measured through older person interviews, structured mealtime observations and activity mapping) at each site.

**Results**

Wards had varied contextual barriers which altered dynamically with time. Two experienced facilitators supported four novice facilitators through interactive training and regular structured reflection, data management, networking and organisational influence. Novice facilitators used many implementation strategies to facilitate 45 discrete improvements at individual, team and system level. Patient interviews (42 before, 38 after) showed better communication about program goals. Observations of 283 meals before and 297 after implementation showed improvements in mealtime positioning and assistance. Activity mapping (85 patients before, 111 after) showed improvements in cognitive and social engagement but inconsistent changes in mobility. Observed improvements are plausible mediators of reduced delirium.

**Conclusions**

A multi-level enabling facilitation approach supported adaptive implementation to varied contexts to support mechanisms of change which partly achieved the programme goals. Contexts changed over time, suggesting the need for adequate time and continued facilitation to embed, enhance and sustain age-friendly practices on acute care wards.

### O34 Exploring sustained receipt of recommended antenatal care for alcohol consumption, by pregnant women following an effective practice change intervention

#### Alix Hall^1^, Emma Doherty^2^, Nicole Nathan^2^, John Wiggers^2^, John Attia^1^, Belinda Tully^2^, Elizabeth Elliott^3^, Christopher Oldmeadow^4^, Simon Chiu^4^, Melanie Kingsland^2^

##### ^1^School of Medicine and Public Health, Faculty of Health and Medicine, University of Newcastle, NSW, Australia; ^2^Hunter New England Population Health, Hunter New England Local Health District, NSW, Australia; ^3^Discipline of Child and Adolescent Health, Faculty of Medicine and Health, The University of Sydney, Sydney, Australia; ^4^Hunter Medical Research Institute, New Lambton Heights, NSW, Australia

*Implementation Science 2023,* **18(2):**O34

**Background**

Sustaining evidence-based care is essential for ensuring population benefits. However, the impact of effective implementation strategies may reduce over time. This study aimed to explore the ongoing receipt of recommended antenatal care for alcohol consumption by pregnant women, following an effective practice change intervention.

**Methods**

This was a secondary analysis of survey data collected from women receiving antenatal care from the largest sector of a randomised stepped-wedge trial. Interrupted time series of data collected across the 31-month study period was used to explore: the rate, time points and extent of change in women’s reported receipt of recommended antenatal care for alcohol consumption, following the delivery of an effective practice change intervention.

**Results**

Data from 4,909 (82% consented) women were analysed. Receipt of recommended care reduced significantly per week for the 16.5 month post- implementation period, for outcomes: assessment of alcohol consumption (-0.66; 95% CI: -1.1, -0.26; p = 0.002), advice not to consume alcohol during pregnancy and of potential risks (-0.63; 95% CI: -1.1, -0.22; p = 0.003), and complete care relevant to alcohol risk level (advice and referral) (-0.64; 95% CI: -1.1, -0.22; p = 0.003). A non-statistically significant reduction was also observed for: all guideline elements relevant to alcohol risk level (-0.36; 95% CI: -0.72, 0.00; p = 0.050). Despite a decrease in women’s receipt of recommended care post-implementation, rates were still higher post-implementation than what was observed pre-implementation.

**Conclusions**

These findings highlight the need for ongoing monitoring of care delivery and the potential need for additional sustainability strategies.

### O35 Reducing the overuse of proven ineffective interventions in infants with bronchiolitis: mixed method study to understand uptake and impact of adaptions to proven implementation strategies

#### Emma Tavender^1,2^, Tim Robertson^3^, Sharon O'Brien^3^, Libby Haskell^4,5^, Rachel Schembri^1^, Franz Babl^1,2,6^, Stuart Dalziel^4,5^, Meredith Borland^3,7^

##### ^1^Murdoch Children's Research Institute, Parkville, VIC, Australia; Departments of Paediatrics and Critical Care, The University of Melbourne, VIC, Australia; ^3^Perth Children's Hospital, Perth, WA, Australia; ^4^Starship Children’s Hospital, Auckland, NZ; ^5^The University of Auckland, Auckland, NZ; The Royal Children’s Hospital, Parkville, VIC, Australia; ^7^School of Medicine, Divisions of Emergency Medicine and Paediatrics, University of Western Australia, WA, Australia

*Implementation Science 2023,* **18(2):**O35

**Background**

A cluster randomised trial in 26 Australasian hospitals found targeted implementation strategies were effective at improving bronchiolitis care and reducing the use of five unnecessary therapies and management processes (salbutamol, adrenaline, corticosteroids, antibiotics and chest x-rays). We aimed to understand the uptake, adaption and impact of these implementation strategies in a “real-world” clinical setting to inform national roll-out.

**Methods**

Implementation strategies were provided to four Western Australian Hospital Emergency Departments (EDs). We conducted a mixed methods process evaluation to track uptake and local adaption processes with adaption tracking forms and qualitative interviews with staff. Descriptive statistical analysis was used for quantitative data. Interview transcripts were coded using thematic content analysis. The Framework for Reporting Adaptions and Modifications-Enhanced (FRAME-I) informed data collection. We evaluated compliance of the 5 recommendations using retrospective data collection of 120 bronchiolitis patients pre-intervention (2019 season) and 120 post-intervention (2021 season).

**Results**

Twelve interviews were held over 2 months. All sites used a range of implementation intervention components including a combination of face-to-face education, audit and feedback and site-specific modifications of the education packages. Modifications included shortening and adjusting content/style of presentations for different target clinician groups. In 2019, adherence to guidelines was 357/457 infants (78.12%) in comparison with 379/443 (85.55%) in 2021, RD 7.43 (95%CI -0.59;15.46).

**Conclusions**

Provision of proven implementation strategies to four EDs resulted in improved bronchiolitis care. Maximising this benefit by providing guidance on how to adapt and effectively use the implementation strategies will enhance knowledge on scaling improvement and sustainable practice change.

### O36 Improving and sustaining advance care planning (ACP) within oncology settings: Using the Theoretical Domains Framework (TDF) to identify the barriers and enablers

#### Lisa Guccione^1^, Sonia Fullerton^1^, Jill Francis^2^

##### ^1^Peter MacCallum Cancer Centre, Melbourne, VIC, Australia; ^2^Melbourne University, Melbourne, VIC, Australia

*Implementation Science 2023,* **18(2):**O36

**Background**

Advance care planning (ACP) is the process of individuals discussing and recording personal values, beliefs, and preferences so that, in the event they lose capacity, a person receives care consistent with their preferences. Despite Department of Health recommendations, national rates of documentation for people with cancer are only 27%, well below the target of 50%. The aim of this study was to identify barriers to ACP across the care pathway, at a world-leading comprehensive cancer centre.

**Methods**

A mixed methods design was used to: 1) identify ACP touchpoints across the care pathway and 2) explore barriers and enablers of ACP. Twenty-two key stakeholders were recruited to the study including staff, and consumers. Two focus groups explored touchpoints and opportunities for ACP across the care pathway, to develop a process map. The “action, actor, context, target, time” (AACTT) Framework was used to specify behaviours. Semi-structured interviews explored barriers and enablers at each touchpoint. The TDF was used to guide the interviews and analysis.

**Results**

Process maps representing differing perspectives between consumers and hospital staff (medical, nursing, allied health, and administrative staff) identified 20 “actions” associated with ACP across the care pathway. The AACTT analysis clarified that 5 staff roles were responsible for performing ACP-related behaviours. Barriers included perceived emotional consequences for patients and inadequate digital infrastructure for accessing ACP documentation at the point of care.

**Conclusions**

Using a theory-based approach, barriers for ACP across the care pathway were identified. To improve ACP uptake in oncology settings, interventions should target these barriers.

### O37 Why is advance care planning underused in oncology settings? Systematic overview of reviews to identify benefits, barriers, enablers, and interventions to improve uptake

#### Lisa Guccione^1^, Sonia Fullerton^1^, Karla Gough^1^, Amelia Hyatt^1^, Sanchia Aranda^1^, Jill Francis^2^

##### ^1^Peter MacCallum Cancer Centre, Melbourne, VIC, Australia; ^2^Melbourne University, Melbourne, VIC, Australia

*Implementation Science 2023,* **18(2):**O37

https://doi.org/10.3389/fonc.2023.1040589

## What works, why and for whom when implementing programs in primary care and community settings?

### O38 Do first impressions of trust predict therapeutic alliance and health outcomes for people presenting with musculoskeletal conditions?

#### Sonia Coates^1^, Kerrie Evans^1^, Claire Ashton-James^2^, Eileen Boyle^3^, Darren Beales^3^, Kwangil Kang^1^, Trudy Rebbeck^1^

##### ^1^Faculty of Medicine and Health, University of Sydney, NSW, Australia; ^2^Pain Management Research Institute, University of Sydney, NSW, Australia; ^3^Faculty of Health Sciences, Curtin University, WA, Australia

*Implementation Science 2023,* **18(2):**O38

**Background**

A positive therapeutic relationship between patients and their health care professionals (HCPs) is associated with improved health outcomes in musculoskeletal conditions however initial perceptions of HCP trustworthiness and their relationship to therapeutic alliance and health outcomes have not been evaluated. The aims of this study were to (i) determine the relationship between initial trust and therapeutic alliance and satisfaction with care at 3 months and; (ii) evaluate the relationship between initial trust and health outcomes at 3 months. Factors predicting perceptions of HCP trustworthiness such as profession and expertise were explored.

**Methods**

Prospective observational study nested within a randomised controlled trial. Participants presenting with low back, neck pain or knee osteoarthritis within 4 weeks of seeking care were eligible. Seven hundred and sixty-six participants completed baseline questionnaires and perceptions of trustworthiness in their self-nominated primary HCP. Outcomes assessed at three months included: therapeutic alliance (WAI-SR), satisfaction with care and health outcomes (GPE and pain-related disability). Associations between variables of interest were assessed using Spearman's Rho correlation coefficient and multivariate linear regression analysis.

**Results**

Initial trust predicted the therapeutic relationship (Adjusted R2=0.16, p<0.001), satisfaction with care (Adjusted R2=0.08, p<0.001), and pain-related disability at 3 months (Adjusted R2=0.01, p=0.003) but not GPE. Perceptions of HCP trustworthiness were high and similar amongst medical and allied health professional groups.

**Conclusions**

Initial perceptions of HCP trustworthiness are moderately associated with trust, therapeutic alliance and satisfaction with care at 3 months and may predict health outcomes.

### O39 The Implementing work-related Mental health guidelines in general Practice (IMPRovE) intervention: Process followed towards planning for sustainability and scale-out

#### Vera da Costa^1^, Samantha Chakraborty^1^, Justin Kenardy^2^, Bianca Brijnath^3^, Duncan Mortimer^1^, Joanne Enticott^1^, Michael Kidd^4^, Lyndal Trevena^5^, Sharon Reid^5^, Alex Collie^1^, Danielle Mazza^1^

##### ^1^Monash University, VIC, Australia; ^2^The University of Queensland, QLD, Australia; ^3^National Ageing Research Institute, VIC, Australia; ^4^Australian National University, ACT, Australia; ^5^University of Sydney, NSW, Australia

*Implementation Science 2023,* **18(2):**O39

**Background**

The IMPRovE trial is a hybrid III trial aiming to implement the “Clinical Guidelines for the diagnosis and management of work-related mental health conditions” in general practice. We applied the RE-AIM framework to design the trial and co-create an intervention that effects clinical and implementation outcomes necessary for achieving sustainability and scalability. This paper describes the process we used to evaluate the delivery of a complex intervention, and to understand which intervention components we should aim to sustain and scale-out.

**Methods**

GPs evaluation of their participation in an academic detailing session was gathered, and engagement activity in the virtual community of practice (VCoP) was tracked over a 72-week period. We used the Realist Evaluation model involving diaries from academic detailers, quantitative descriptive analysis of engagement activity by participants on the VCoP (tracked over a 72-week period), qualitative interviews with GP and patient participants to examine context, mechanism, and outcomes.

**Results**

We linked constructs of normalisation process theory with the Context-Mechanism-Outcome framework of realist evaluation, to evaluate how the intervention was implemented and adopted by GPs. We will review these outcomes in an upcoming stakeholder forum and prioritise aspects of the intervention that are amenable for scale-up or sustainability.

**Conclusions**

VCoPs and delivery of academic detailing are a novel and an increasingly popular learning vehicles for general practitioners. It is necessary to begin planning for sustainability and scale-out at the outset of a project in order to embed processes that can subsequently inform assessment of sustainability and scale-out.

### O40 Using a reverse translation approach to identify factors contributing to sustainable adoption of peer support programs after brain injury

#### Marlena Klaic, Lauren Kosta

##### University of Melbourne, VIC, Australia

*Implementation Science 2023,* **18(2):**O40

**Background**

The annual incidence of traumatic brain injury (TBI) in Australia is estimated to be 200,000, of which 10 percent will have a moderate to severe injury affecting cognition and function. Peer support programs offer survivors of brain injury with the opportunity to develop self-management skills as they learn from and with others living with a TBI. Brain Injury Matters (BIM) have been the leading provider of community-based peer support programs for several years in Victoria. Attendance rates are consistently high, yet little is known about the factors contributing to this sustainable intervention. We used a reverse translation approach to explore how and why community-based peer support programs for survivors of TBI are successful. Reverse translation begins with a real-life clinical experience and works backward to identify factors influencing uptake.

**Methods**

Data were collected from six groups, including one online platform using a mixed-methods approach consisting of semi-structured interviews and questionnaires based on the theoretical framework of acceptability and audit of routinely collected data.

**Results**

We found that acceptability of community-based peer support programs for survivors of TBI is high, particularly for perceived effectiveness, affective attitude and ethicality.

**Conclusions**

A reverse translation approach has the benefit of identifying factors that may impact on sustainable adoption of an intervention but are not usually captured in a clinical trial. This study found that peer support groups after a TBI are largely acceptable to both those providing and those receiving the intervention which we hypothesise is associated with feasibility and sustainability.

### O41 Identifying healthy eating and physical activity evidence-practice gaps in early childhood education and care services across Australia: A cross-sectional study

#### Melanie Lum^1,2^, Sze Lin Yoong^2,3^, Luke Wolfenden^1,2^, Alice Grady^2,4^, Jannah Jones^2,4^

##### ^1^School of Medicine and Public Health, University of Newcastle, NSW, Australia; ^2^National Centre of Implementation Science, University of Newcastle, NSW, Australia; ^3^Global Obesity Centre, Institute for Health Transformation, School of Health and Social Development, Deakin University, VIC, Australia; ^4^Hunter New England Population Health, Hunter New England Local Health District, NSW, Australia

*Implementation Science 2023,* **18(2):**O41

**Background**

Early childhood education and care (ECEC) services are a key setting to improve healthy eating- and physical activity-related behaviours of children. Several practices in ECEC have demonstrated effectiveness in improving children’s diet and physical activity (e.g. provision of various healthy eating education regularly). The current implementation of these practices across Australia is unknown. This study aims to describe the prevalence of implementation of evidence-based healthy eating and physical activity practices in ECEC services nationally and examine differences in the prevalence of implementation of these practices by service characteristics.

**Methods**

2,050 centre-based ECEC services across Australia were randomly selected and invited to complete a survey via telephone or online (August 2021-April 2022). Service characteristics and implementation of 18 evidence-based healthy eating and physical activity practices were assessed using items based on valid and reliable tools. Linear mixed regression analyses were performed to examine differences in implementation by service characteristics.

**Results**

993 (51.3%) eligible services consented to participate. Less than 18% of services provided training for either heathy or physical activity. Preliminary analyses indicate less than 50% of services are implementing 10 of the 18 practices assessed. Association analyses are expected to be completed early 2023.

**Conclusions**

The findings of this study demonstrate the current prevalence of implementation of evidence-based healthy eating and physical activity practices in ECEC services, and will identify differences in implementation rates by service characteristics. This study highlights several evidence-practices gaps, such as educator training, indicating that additional implementation support may be warranted for these practices.

### O42 Scaling What Works: Protocol for evaluating the scalability of 16 programs designed to improve the mental health and wellbeing of men and boys

#### Anna Williamson, Chloe Ang, Chloe Jacobs

##### Centre for Evidence and Implementation, VIC, Australia

*Implementation Science 2023,* **18(2):**O42

**Background**

Scaling What Works (SWW) is a funding program established by Movember to support the ongoing scaling, development, implementation, and evaluation of prevention and/or early intervention programs that have already shown promise in improving mental health and/or suicide prevention outcomes for boys and men across Australia, Canada, the United Kingdom, and Ireland.

**Methods**

A mixed methods evaluation has been developed to assess the impact and cost effectiveness of the projects funded as part of Scaling What Works, on both a project and a fund-level, and the implementation of each project. The Intervention Scalability Assessment Tool will be used as a framework to answer all evaluation questions and systematically assess the scalability of each of the funded projects over a two-year period.

**Results**

Sixteen projects were funded as part of Scaling What Works. Projects differed considerably in terms of target group, setting, scale, intervention, extent of planned adaptation, and place on the translational research pathway, necessitating a structured yet flexible approach to evaluation.

**Conclusions**

Scaling What Works will provide a unique opportunity to systematically explore the factors associated with scalability across 16 diverse projects aiming to improve the mental health of men and boys.

### O43 Using the Implementation Research Logic Model (IRLM) to guide implementation and evaluation of eConsultant in Queensland

#### Jenny Job^1^, Maria Donald^2^, Breanna Lepre^1^, Caroline Nicholson^1^, Claire Jackson^1^

##### ^1^Centre for Health System Reform and Integration, Mater Research, University of Queensland, QLD, Australia; ^2^General Practice Clinical Unit, Faculty of Medicine, The University of Queensland, QLD, Australia

*Implementation Science 2023,* **18(2):**O43

**Background**

eConsultant provides specialist input for general practitioners (GPs) via secure-messaging within 3-business days to support care for adult patient who would otherwise require an outpatient referral. GPs send a Request-for-Advice to the eConsultant and discuss the eConsultant advice at patient follow-up. Our evaluation of the implementation of eConsultant service in two Australian regions (Western Queensland, Brisbane South) is guided by the Implementation Research Logic Model (IRLM).

**Methods**

Our prospective mixed-methods observational study included tracking implementation activities and outcomes (effectiveness/adoption). Semi-structured interviews conducted with GPs/stakeholders to understand determinants of implementation, were analysed thematically guided by the Consolidated Framework for Implementation Research. Implementation activities were coded against 73 Expert Recommendations for Implementing Change implementation strategies. Adoption (enrolment/usage by GP practices/GPs) and effectiveness (time to specialist input) were assessed.

**Results**

To date 22 GP practices have completed enrolment in the program and 60 GPs have sent a total of 180 requests-for-advice (1.6 days mean specialist response time). Implementation was guided by 15 different implementation strategies. Qualitative interviews, conducted with 11 GPs and 4 stakeholders identified barriers to using eConsultant related to secure-messaging access, reliance on existing referral options and workforce issues. Key facilitators identified were engaging GPs, the positive response from patients to the program, and the relative advantage of eConsultant over other options.

**Conclusions**

IRLM enabled systematic use of frameworks which highlighted priorities for successful implementation state-wide including an improvement in secure-messaging access and operability in some settings and offering a greater variety of specialties to embed the eConsultant option in GP advice processes.

### O44 Implementation of a GP:Physiotherapist partnership in primary care for chronic obstructive pulmonary disease improves case finding and evidence-based management

#### Lisa Pagano^1^, Zoe McKeough^1^, Sally Wootton^2^, Andrew SL Chan^3,4^, Sriram Mahadev^3,4^, Nick Zwar^5^, Deborah Pallavacini^6^, Sarah Dennis^1,7,8^

##### ^1^Faculty of Medicine and Health, The University of Sydney, NSW, Australia; ^2^Chronic Disease Community Rehabilitation Service, Northern Sydney Local Health District, NSW, Australia; ^3^Department of Respiratory and Sleep Medicine, Royal North Shore Hospital, NSW, Australia; ^4^Northern Clinical School, Faculty of Medicine and Health, University of Sydney, Sydney, Australia; ^5^Faculty of Health Sciences and Medicine, Bond University, QLD, Australia; ^6^Sydney North Primary Health Network, NSW, Australia; ^7^Ingham Institute for Applied Medical Research, Sydney, Australia; ^8^South Western Sydney Local Health District, Liverpool, Australia

*Implementation Science 2023,* **18(2):**O44

**Background**

The aim of this study was to evaluate whether the implementation of a General Practice (GP): physiotherapist partnership model of care improves the diagnosis and management of COPD in primary care by improving implementation of key management guidelines.

**Methods**

A pre/post study was conducted including four general practices. Pre/post-bronchodilator spirometry was performed by a cardiorespiratory physiotherapist placed in each general practice on “at risk” participants (aged >40 years, current/ex-smoker) or people with “existing” COPD. For those with confirmed airflow obstruction on spirometry (FEV_1_/FVC < 0.7), a management plan underpinned by evidence-based guidelines was implemented by the physiotherapist in collaboration with the GP including physical activity counselling, smoking cessation advice, referral to pulmonary rehabilitation (PR), initiation of a COPD action plan and GP referral for medical management. Intervention occurred at baseline, one-month, and 3-months.

**Results**

148 participants (mean age 70 years (SD 11.1), 57% female) attended a baseline assessment (117 “at risk”, 31 “existing” COPD) from 748 people invited. Obstruction was confirmed in 17% of “at risk” and 77% of “existing” COPD [1]. The physiotherapist correctly classified the level of obstruction in 98.6% of cases [1]. Of those with airflow obstruction, all participants had smoking cessation interventions initiated, 78% (21/27) were referred to PR and 87% had an action plan initiated.

**Conclusions**

This GP: physiotherapist model of care indicates a rate of case finding similar to other studies and has the potential to improve early identification through case finding and implementation of evidence-based management components according to guidelines in primary care.

**Reference**

1. Pagano L, Dennis S, Wootton S, et al. Identifying airway obstruction in primary care: is there a role for physiotherapists? BMC Prim Care. 2022 Dec 14;23(1):324.

